# Explicit solutions to correlation matrix completion problems, with an application to risk management and insurance

**DOI:** 10.1098/rsos.172348

**Published:** 2018-03-14

**Authors:** Dan I. Georgescu, Nicholas J. Higham, Gareth W. Peters

**Affiliations:** 1 Prudential Regulation Authority, Bank of England, London EC2R 6DA, UK; 2 School of Mathematics, University of Manchester, Manchester M13 9PL, UK; 3 Department of Actuarial Mathematics and Statistics, Heriot-Watt University, Edinburgh EH14 4AS, UK

**Keywords:** matrix completion, correlation matrix, positive definite matrix, maximal determinant, chordal graph, risk management

## Abstract

We derive explicit solutions to the problem of completing a partially specified correlation matrix. Our results apply to several block structures for the unspecified entries that arise in insurance and risk management, where an insurance company with many lines of business is required to satisfy certain capital requirements but may have incomplete knowledge of the underlying correlation matrix. Among the many possible completions, we focus on the one with maximal determinant. This has attractive properties and we argue that it is suitable for use in the insurance application. Our explicit formulae enable easy solution of practical problems and are useful for testing more general algorithms for the maximal determinant correlation matrix completion problem.

## 1. Introduction

In many applications, missing values in a set of variables lead to the construction of an approximate correlation matrix that lacks definiteness and hence is not a true correlation matrix. Replacing the approximate correlation matrix by the nearest correlation matrix is a popular way to restore definiteness, and good numerical methods are available for this task [1–4]. Here we are concerned with problems in which the missing values are in the correlation matrix itself. Some of the matrix entries are known, having been estimated, prescribed by regulations or assigned by expert judgement, but the other entries are unknown. The aim is to fill in the missing entries in order to produce a correlation matrix. Of course there are, in general, many possible completions. For example, the partially specified matrix $A=[\begin{array}{cc} 1 & a_{12}\\ a_{12} & 1 \end{array}]$ is a correlation matrix for any *a*_12_ such that |*a*_12_|≤1. Our focus is on the completion with maximal determinant (given by *a*_12_=0 in this example), which is unique when completions exist.

This work is motivated by an application in the insurance industry, where a correlation matrix is used in the aggregation of risk exposures required by industry regulations. Correlations are particularly likely to be missing in areas of risk management and insurance where data and loss event history is scarce and so there are large gaps in the data records, such as in operational risk, reinsurance, catastrophe insurance, life insurance and cyber risk. The estimation of missing correlations is also important in banking capital calculations, for example, in the internal model (IM)-based approach to market risk and the advanced measurement approach for operational risk.

We give explicit solutions for the maximal determinant completion problem with some practically occurring block structures. The solutions are implicit in the literature but have not previously been given in the form of explicit matrix expressions that are readily translated into code. Underlying the solutions is a duality between the completion problem and the covariance selection problem, the consequence of which is that the required completion is characterized by having zero elements in its inverse in the positions corresponding to the unknown elements of the original matrix.

In the next section, we describe the insurance regulation application and explain why the maximal determinant completion is appropriate. We give explicit solutions in §4 for matrices with certain practically important block 3×3 and 4×4 structures. In §5, we give a numerical example in which we compare the maximal determinant completion with the nearest correlation matrix and show its use with the shrinking method of Higham *et al.* [5]. In §6, we show how to deal with larger block structures. Concluding remarks are given in §7.

## 2. Insurance application

Calculations of the capital that financial firms are required to hold can allow for some diversification between types of risks. Diversification can be derived from the dependency relations between capital for individual risks, specified in the form of a correlation matrix. For example, European insurers subject to the Solvency II Directive [6] are allowed to take diversification effects into account when calculating their solvency capital requirement (SCR). The SCR is defined as the value at risk of the surplus of assets over liabilities of an insurance undertaking subject to a confidence level of 99.5% over a 1-year period. The standard formula (SF) for aggregating the capital requirement for different risk exposures is the square root of a linear function of the correlation matrix *Σ* specifying the dependence between them: $\sqrt{v^{T}\Sigma v}$, where *v* is a vector of capital requirements for the individual risk category. The assumption is that the underlying distribution of risk capital is multivariate normal, or more generally elliptically contoured.

However, it is often the case that not all of the entries in the correlation matrix *Σ* are known. For example, the insurer may be exposed to different risks than those considered in the SF, or may not be using the SF at all. The question arises of how to specify the dependency relations between the risks. Generally, some of the individual correlation coefficients are known because they have been estimated with reasonable confidence from data, specified by regulations (as in the case of the SF) or derived by expert judgement. However, the firm may be modelling a risk exposure not considered by the SF that is present in one business unit but not in another (a business unit-specific or BU-specific risk). This is particularly relevant where the insurance group operates in many different countries and underwrites different risks, has insufficient data to reliably estimate a correlation and has insufficient expertise to set the assumption by expert judgement. The problem is to complete a partial correlation matrix with a particular pattern of unspecified entries. This same problem arises in banking capital calculations. It is worth remarking that without formal matrix completion methods such as the one developed here, the heuristic approaches that are currently adopted in practice can be subject to moral hazard, where a firm may be incentivized by a capital reduction to perform matrix completions which increase diversification gains, thereby reducing the required capital they must hold.

Correlation coefficients are typically fully specified in the business unit with the BU-specific risk. Correlations are also specified between similar risk families in different business units. For example, in table 1, which illustrates the case of just two business units, both are exposed to risks *x* and *y*, but only BU_1_ is exposed to risk *z*. Correlations are specified between risks *z*, *x* and *y* in BU_1_, but not between *x* and *y* in BU_2_ and *z* in BU_1_. This is a simplified example used for illustrative purposes only. In a more complex case, the second business unit would also have a BU-specific risk. In the most general case, there are many business units with many BU-specific risks as well as different numbers of risk families, and the matrices involved can have hundreds of columns.

**Table 1.** Example with two business units where correlations are not specified between risk *z* in BU_1_ and the risks *x* and *y* in BU_2_.

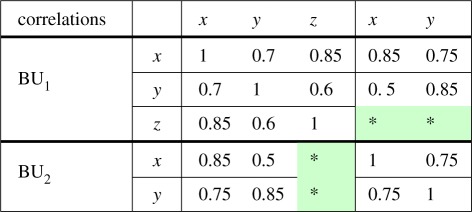

We need to complete the partial correlation matrix $\bar{\Sigma }$ to a fully specified correlation matrix, that is, as the diagonal is fully specified as ones, to a positive-definite matrix. Many completions are possible, which introduces uncertainty around the range of potential capital outcomes. The completion of most interest is usually a best-estimate completion in some sense. A good candidate is that completion which has maximum determinant, denoted by MaxDet. MaxDet has several useful theoretical properties.

(i) *Existence and uniqueness*: if positive semi-definite completions exist, then there is exactly one MaxDet completion [7].
(ii) *Maximum entropy model*: MaxDet is the maximum entropy completion for the multivariate normal model, where maximum entropy is a principle of favouring the simplest explanations. In the absence of other explanations, we should choose this principle for the null hypothesis in Bayesian analysis [8].
(iii) *Maximum-likelihood estimation*: MaxDet is the maximum-likelihood estimate of the correlation matrix of the unknown underlying multivariate normal model.
(iv) *Analytic centre*: MaxDet is the analytic centre of the feasible region described by the positive semi-definiteness constraints, where the analytic centre is defined as the point that maximizes the product of distances to the defining hyperplanes [9].

Properties (i)–(iii) above are discussed in the context of the covariance selection problem by Dempster [10]. We note that the determinant of a correlation matrix is at most 1, as can be seen by applying Hadamard’s inequality [11], Thm. 7.8.1.

**Figure 1.**
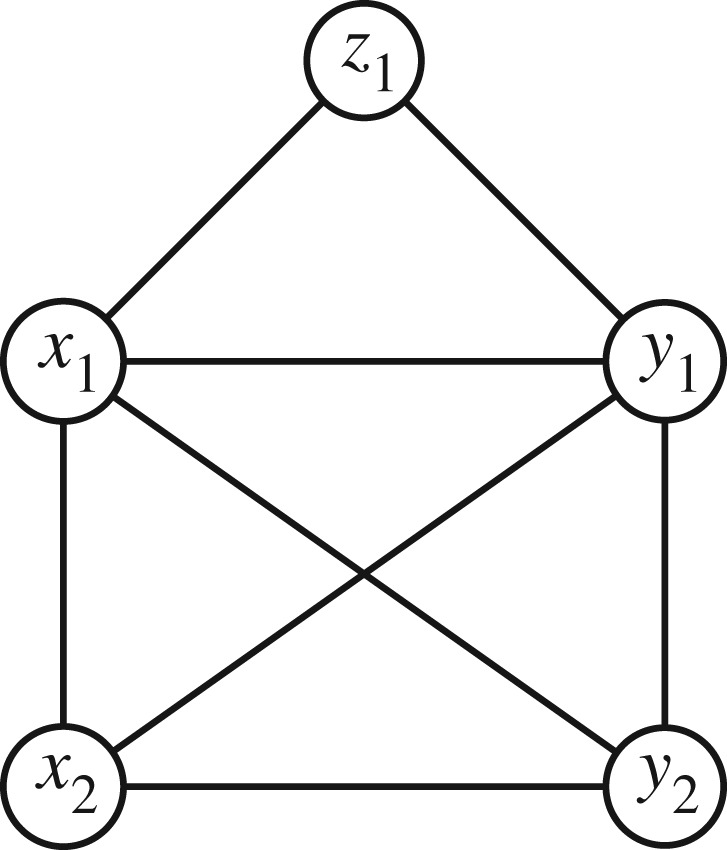
Chordal graph corresponding to the example with only one BU-specific risk in table 1.

Grone *et al.* [7] show that a partially specified Hermitian matrix with specified positive diagonal entries and positive principal minors (where specified) can be completed to a positive-definite matrix regardless of the values of the entries if and only if the undirected graph of the specified entries (ignoring the leading diagonal) is chordal. A graph is chordal if every cycle of length greater than or equal to 4 has a chord, which is an edge that is not part of the cycle but connects two vertices of the cycle. If the graph is not chordal, then whether a positive semi-definite completion exists depends on the specified entries. It is straightforward to show that all the sparsity patterns considered in this paper are chordal, and therefore a positive semi-definite completion is possible. For example, the adjacency graph for the case in table 1 is shown in figure 1.

Grone *et al.* [7] show, additionally, that if a positive-definite completion exists then there is a unique matrix in the class of all positive-definite completions whose determinant is maximal. See also Johnson [12] for a survey of these and related results.

As we will be dealing with large matrices with block patterns of specified and unspecified entries, it is convenient to introduce the definition of a ‘block chordal’ graph equivalent to the above. A block is a subgraph which is complete in terms of edges (a clique). Two blocks are connected by an edge if every vertex has an edge to every other vertex, so the two blocks considered together also form a clique. A graph is block chordal if every cycle of blocks of length greater than or equal to 4 has a chord. Finally, a block chordal graph is also chordal because every block is either fully specified or fully unspecified, so collapsing each block into one node means that we do not lose any information in the graph.

## 3. Dual problems

Dempster [10] proposes a related problem, covariance selection, and Dahl *et al.* [13] and Vandenberghe *et al.* [9] show that MaxDet completion and covariance selection are duals of each other. Covariance selection aims to simplify the covariance structure of a multivariate normal population by setting elements of the inverse of the covariance matrix to zero. The statistical interpretation is that certain variables are set to be pairwise conditionally independent. For random variables *a*, *b*, *c*, the variables *a* and *b* are conditionally independent given *c* if

3.1 f(a | b,c)=f(a | c).

In other words, once we know *c*, knowledge of *b* gives no further information about *a*. In a multivariate normal setting, (3.1) is equivalent to the inverse of the covariance matrix for those three variables having zero in the position corresponding to the covariance between *a* and *b*. To see this, in general, partition a multivariate normal random variable *X* into two sets: *I* and *J* (the idea being that the *I* variables are independent of each other, conditioning on *J*). The conditional distribution of *X*_*I*_ given *X*_*J*_ is shown in [14], Thm. 2.5.1 to be *normal* with covariance matrix

$$\Sigma_{I\;|\;J}=\Sigma_{II}-\Sigma_{IJ}\Sigma_{JJ}^{-1}\Sigma_{JI}.$$

Conditional independence means that $\Sigma_{II}-\Sigma_{IJ}\Sigma_{JJ}^{-1}\Sigma_{JI}$ is diagonal, i.e. that *X*_*i*_ and *X*_*j*_ are conditionally independent for *I*=(*i*,*j*). The expression for *Σ*_*I* | *J*_ is identical to the inverse of the Schur complement of *Σ*_*JJ*_ in *Σ*:

$${\left(\Sigma^{-1}\right)}_{II}={\left[\begin{array}{cc} \Sigma_{II} & \Sigma_{IJ}\\ \Sigma_{JI} & \Sigma_{JJ} \end{array}\right]}_{II}^{-1}={\left(\Sigma_{II}-\Sigma_{IJ}\Sigma_{JJ}^{-1}\Sigma_{JI}\right)}^{-1}.$$

Therefore we require this block to be diagonal or (*Σ*^−1^)_*ij*_=0 for *i*,*j*∈*I* with *i*≠*j*.

Another way to see that a determinant-maximizing completion must have zeros in the inverse corresponding to the free elements of *Σ* is by a perturbation argument. We need the following lemma [15], Lem. 26.

**Lemma 3.1**

*For* $v,w,x,y\in R^{n}$,

$$\det(I+vx^{T}+wy^{T})=(1+v^{T}x)(1+w^{T}y)-(v^{T}y)(w^{T}x).$$

Using the lemma, we consider how the determinant of a symmetric positive-definite matrix $A\in R^{n\times n}$ changes when we perturb *a*_*ij*_ (and *a*_*ji*_, by symmetry). Let

$$A(\epsilon )=A+\epsilon (e_{i}e_{j}^{T}+e_{j}e_{i}^{T}),$$

where *e*_*i*_ is the *i*th column of the identity matrix. Let *B*=*A*^−1^ and partition *B*=[*b*_1_,…,*b*_*n*_]. Applying the lemma, we have

$$\begin{array}{rl} \det A(\epsilon ) & \;=\det(A(I+\epsilon (b_{i}e_{j}^{T}+b_{j}e_{i}^{T})))\\ & \;=\det(A)\;\det(I+\epsilon (b_{i}e_{j}^{T}+b_{j}e_{i}^{T}))\\ & \;=\det(A)[(1+\epsilon b_{i}^{T}e_{j})(1+\epsilon b_{j}^{T}e_{i})-\epsilon^{2}(b_{i}^{T}e_{i})(b_{j}^{T}e_{j})]\\ & \;=\det(A)[(1+\epsilon b_{ji})(1+\epsilon b_{ij})-\epsilon^{2}b_{ii}b_{jj}]\\ & \;=\det(A)(1+2\epsilon b_{ij}+\epsilon^{2}(b_{ij}^{2}-b_{ii}b_{jj})). \end{array}$$

We want to know when detA(0) is maximal. As

$$\frac{d}{d\epsilon }\;\det A(\epsilon ){|}_{\epsilon =0}=2\;\det(A)b_{ij},$$

we need *b*_*ij*_=0 for a stationary point at *ϵ*=0, and from

$$\frac{d^{2}}{d\epsilon^{2}}\;\det A(\epsilon ){|}_{\epsilon =0}=2\;\det(A)(b_{ij}^{2}-b_{ii}b_{jj})<0$$

(because *B* is positive definite), we see that when *b*_*ij*_=0, the quadratic function detA(ϵ) has a maximum at *ϵ*=0.

## 4. Maximal determinant completions

In general, solving the MaxDet completion problem (or, equivalently, the covariance selection problem) requires solving a convex optimization problem on the set of positive-definite matrices [13]. We wish to obtain explicit, easily implementable solutions for some practically important cases arising in the insurance application. Such solutions are helpful for practitioners and also useful for testing algorithms that tackle the most general problem.

Let *Σ* denote the solution of the MaxDet completion problem for the partially specified correlation matrix $\bar{\Sigma }$. We give a result for an L-shaped pattern of unspecified entries. Note that we do not require a unit diagonal in theorem 4.1, so it applies more generally than just to correlation matrices.

**Theorem 4.1**

*Consider the symmetric matrix*

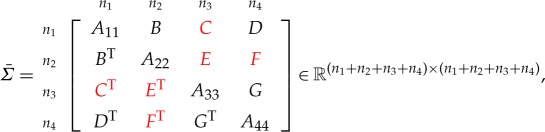

*where C, E and F are unspecified, the diagonal blocks A*_*ii*_*, i=1: 4 are all positive definite and all specified principal minors are positive. The maximal determinant completion is*

4.1 $$C=DA_{44}^{-1}G^{T},\;F=B^{T}A_{11}^{-1}D\;\mathrm{and}\;E=FA_{44}^{-1}G^{T}.$$

**Proof.**

The result can be derived by permuting $\bar{\Sigma }$ so that the unspecified matrices appear in the block (1,3), (1,4) and (2,4) positions, and then applying the results of Dym & Gohberg [16] on completion of block-banded matrices.

The result can also be obtained from [17, Cor. 4.4], in which the unspecified elements of the MaxDet completion are given element-wise in terms of the clique paths in the graph of the specified elements.

Alternatively, an elementary proof based on Gaussian elimination, using the property that *Σ*^−1^ will contain zeros in the positions of the unspecified entries in $\bar{\Sigma }$, is given in [18]. ▪

For the best accuracy and efficiency the formulae (4.1) should be evaluated as follows, avoiding explicit computation of matrix inverses [19]. Compute Cholesky factorizations $A_{11}=R_{11}^{T}R_{11}$ and $A_{44}=R_{44}^{T}R_{44}$; then evaluate

$$C=(DR_{44}^{-1})(R_{44}^{-T}G^{T}),\;F=(B^{T}R_{11}^{-1})(R_{11}^{-T}D)\;\mathrm{and}\;E=(FR_{44}^{-1})(R_{44}^{-T}G^{T}).$$

Each of the terms in parentheses should be evaluated as the solution of a triangular linear system with multiple right-hand sides, and the term $R_{44}^{-T}G^{T}$ can be calculated once and reused.

We identify two useful special cases of theorem 4.1. Both of these are equivalent to [20, Cor. 3.4].

**Corollary 4.2**

*Consider the symmetric matrix*

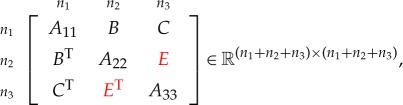

*where* *E* *is unspecified, all the diagonal blocks are positive definite and all specified principal minors are positive. The maximal determinant completion is* $E=B^{T}A_{11}^{-1}C$.

**Proof.**

The result is obtained by setting *n*_3_=0 in theorem 4.1. ▪

The following corollary also appears in [21], Thm. 2.2.3.

**Corollary 4.3**

*Consider the symmetric matrix*

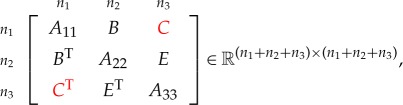

*where* *C* *is unspecified, all the diagonal blocks are positive definite and all specified principal minors are positive. The maximal determinant completion is* $C=BA_{22}^{-1}E$.

**Proof.**

The result is obtained by permuting the matrix to put the unspecified block in the (2,3) block position and then applying corollary 4.2. ▪

Now we consider a pattern of unspecified elements that arises when (for example) an insurance company has four business units where correlations between BU-specific risks are known (described by the specified blocks *A*_11_, *A*_22_, *A*_33_ and *A*_44_) and all the correlations are known for the first group of risks (for example, risk drivers such as interest rates or exchange rates). So here we have a complete first block row and column, and this case cannot be obtained by permuting rows and columns in theorem 4.1.

**Theorem 4.4**

*Consider the symmetric matrix*

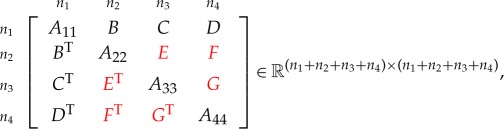

*where E, F and G are unspecified, all the diagonal blocks are positive definite and all specified principal minors are positive. The maximal determinant completion of the matrix is*

$$E=B^{T}A_{11}^{-1}C,\;F=B^{T}A_{11}^{-1}D\;\mathrm{and}\;G=C^{T}A_{11}^{-1}D.$$

**Proof.**

Barrett *et al.* [22] show that the MaxDet completion can be found by a sequence of one-dimensional maximizations on subproblems generated from a chordal ordering. The chordal ordering begins with the graph *G*_0_ of the specified entries. To generate *G*_*k*_ from *G*_*k*−1_, it adds an edge (*i*_*k*_,*j*_*k*_) corresponding to an unspecified entry *a*_*i*_*k*_,*j*_*k*__ to obtain a new chordal graph *G*_*k*_, continuing in this way until all unspecified entries have been added. For the *k*th graph *G*_*k*_, a one-dimensional MaxDet completion is computed for the problem corresponding to the maximal clique of *G*_*k*_ containing the edge (*i*_*k*_,*j*_*k*_).

The graph of the specified entries of our matrix is block chordal and we can complete it by adding the 2–3 edge and completing the leading 3×3 block submatrix by using corollary 4.2 to determine *E*; adding the 3–4 edge and completing the submatrix at the intersection of block rows and columns 1, 3 and 4 by using corollary 4.2 to determine *G*; and finally adding the 2–4 edge and obtaining *F* from theorem 4.1. ▪

Finally, we consider the case where *C*, *E* and *F* are unspecified, and *B* and *G* are partly specified. This result will be needed in §6.

**Theorem 4.5**

*Consider the symmetric matrix*

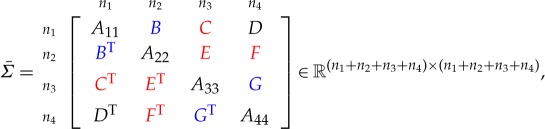

*where C, E and F are unspecified, B and G are partly specified (possibly fully unspecified), all the diagonal blocks are positive definite, all specified principal minors are positive, and the graph of the specified entries is block chordal. If B and G are fully unspecified, then the maximal determinant completion of the matrix is*
4.2
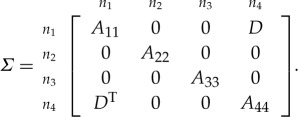

*Otherwise, the maximal determinant completion of B and G is independent of the entries in D.*

**Proof.**

First, consider the case where *B* and *G* are fully unspecified. The graph of the specified entries is trivially block chordal, so a unique determinant-maximizing positive-definite completion exists. The inverse of *Σ* in (4.2) is

$$\Sigma^{-1}=\left[\begin{array}{cccc} A_{11}^{-1}+A_{11}^{-1}DS^{-1}D^{T}A_{11}^{-1} & 0 & 0 & -A_{11}^{-1}DS^{-1}\\ 0 & A_{22}^{-1} & 0 & 0\\ 0 & 0 & A_{33}^{-1} & 0\\ -S^{-1}D^{T}A_{11}^{-1} & 0 & 0 & S^{-1} \end{array}\right],$$

where $S=A_{44}-D^{T}A_{11}^{-1}D$. This is easily seen to be positive definite, and it has zeros in the locations corresponding to the unspecified entries of $\bar{\Sigma }$. Therefore, *Σ* is the maximum determinant completion.

It can be shown that, for the matrix $\bar{\Sigma }$, a chordal ordering, as described in the proof of theorem 4.4, can first take the edges corresponding to the unspecified entries in *B* and *G* before taking those corresponding to *C*, *E* and *F*. Therefore, the one-dimensional maximizations described in the proof of theorem 4.4 that determine the unspecified entries in *B* and *G* are independent of *D*. ▪

We note that our assumption on the positive definiteness of the diagonal blocks is essential to the results. In the insurance application this assumption is satisfied, because firms replace a diagonal block by a positive-definite correlation matrix (typically the nearest correlation matrix subject to a positive lower bound on the smallest eigenvalue) if it is found not to be positive definite.

## 5. Numerical example

The example in table 1 can be completed using corollary 4.2. For a more complex example, consider the case in table 2 where an insurer needs to complete a correlation matrix to integrate different businesses and risks.^1^ This case also arises in the context of Solvency II [6], where a firm has a partial IM composed of

— an IM module,
— some complete SF modules, and
— an incomplete SF module (market risk) where one or more of the submodules have been modelled internally.

**Table 2.** Example of partial internal model Integration Technique 2, where one of the constituents of the standard formula (SF) market risk module (currency risk) has been included in the IM, so correlations are required between the SF market risk submodules and the other SF modules (that is, the green starred cells).

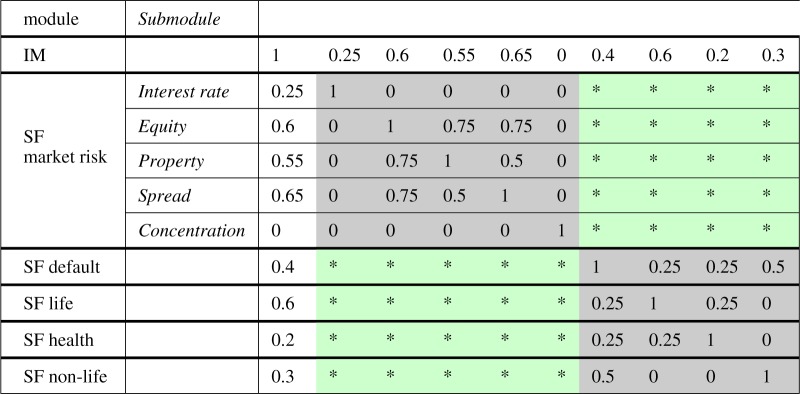

The correlations between the SF elements (grey cells) are specified by regulations, and the firm has calculated some coefficients (white cells) but needs to complete the green entries according to one of the prescribed integration techniques. One of the prescribed integration techniques for completing the missing entries requires two steps: first, determining appropriate upper and lower bounds (based on the firm’s risk profile) for the missing correlations and second, an optimization step to find the completion such that no other set of correlation coefficients results in a higher SCR, while keeping the matrix positive semi-definite (see Solvency II Delegated Regulation ((EU) 2015/35) Annex XVIII(C) [23], also known as Integration Technique 2, IT2). An application of corollary 4.2 can be used as part of the first step to give a central completion (in the sense of property 4 in §2), before other considerations are used to determine the bounds.

The MaxDet completion assigns to the missing submatrix the matrix (in the notation of corollary 4.2)

$$E=\left[\begin{array}{cccc} 0.1000 & 0.1500 & 0.0500 & 0.0750\\ 0.2400 & 0.3600 & 0.1200 & 0.1800\\ 0.2200 & 0.3300 & 0.1100 & 0.1650\\ 0.2600 & 0.3900 & 0.1300 & 0.1950\\ 0 & 0 & 0 & 0 \end{array}\right]$$

(as printed to four decimal places by Matlab), for which ∥*E*∥_*F*_=8.6364×10^−1^ and the determinant and the eigenvalues of the completed matrix *Σ* are, respectively, 2.7348×10^−2^ and


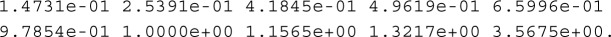


As a check, we compute the norm of the (2: 6,7: 10) submatrix of *Σ*^−1^, which we know should be zero for the MaxDet completion. It evaluates as exactly zero (more typically it will be of the order of 10^−16^, the level of the unit roundoff).

For comparison, let $\tilde{\Sigma }$ denote the matrix obtained from $\bar{\Sigma }$ by setting the unspecified elements to zero. This matrix has smallest eigenvalue −9.9305×10^−3^. We used the algorithm of Higham & Strabić [3]^2^ to compute the nearest correlation matrix to $\tilde{\Sigma }$ in the Frobenius norm, subject to the specified elements of the matrix being fixed. The solution has the completed block

$$E=\left[\begin{array}{cccc} 0.0022 & 0.0084 & 0.0004 & 0.0035\\ 0.0003 & 0.0011 & 0.0001 & 0.0005\\ 0.0025 & 0.0098 & 0.0005 & 0.0040\\ 0.0042 & 0.0164 & 0.0008 & 0.0067\\ 0.0000 & -0.0000 & -0.0000 & 0.0000 \end{array}\right]$$

(the elements in the last row are all of order 10^−16^) and ∥*E*∥_*F*_=2.3216×10^−2^, and it has determinant −1.5653×10^−16^ and eigenvalues


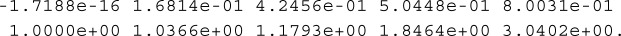


(The non-zero determinant and the negative eigenvalues are a result of rounding errors in the computations, since the exact nearest correlation matrix is singular.)

Another possible use of the MaxDet solution *E* is to compute the smallest *α*∈[0,1] such that *αE* yields a positive semi-definite completion, or equivalently the smallest *α*∈[0,1] such that $(1-\alpha )\tilde{\Sigma }+\alpha \Sigma$ is positive semidefinite. This is precisely the method of shrinking [5] with initial matrix $\tilde{\Sigma }$ and target matrix the MaxDet completion. The optimal *α* is^3^ α_*_=3.4908×10^−2^; it gives ∥α_*_E∥_*F*_=3.0148×10^−2^ and a completion with determinant 3.3809×10^−16^ and eigenvalues


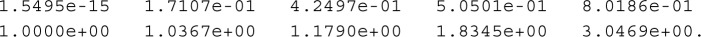


This comparison emphasizes that the MaxDet completion is very different from the nearest correlation matrix, and that through shrinking, it can yield a completion not much further from $\tilde{\Sigma }$ than the nearest correlation matrix.

## 6. Extension to larger block structures

We now present an extension of theorem 4.1 to larger block structures, corresponding to applications with many business units with many BU-specific risks. Correlations are assumed to be known between all ‘standard’ risk drivers, typically the market risks in all business units. This is because there is generally sufficient data to calculate correlations between equity indices, interest rates and credit spreads, say, across economies.

The extension relies on the observation that if the *B* or *G* blocks in theorem 4.1 have unknown entries, then the maximal determinant completions for these blocks are independent of the other entries in the matrix, as shown by theorem 4.5.

Theorem 6.1 shows the calculation for four business units, laid out as two instances of the case in theorem 4.1, in the upper left and bottom right corners of the matrix $\bar{\Sigma }$. Three business units can be obtained as a special case where one business unit has empty elements. More than four business units can be accommodated by repeated applications of theorem 4.1.

**Theorem 6.1**

*Consider the symmetric matrix*

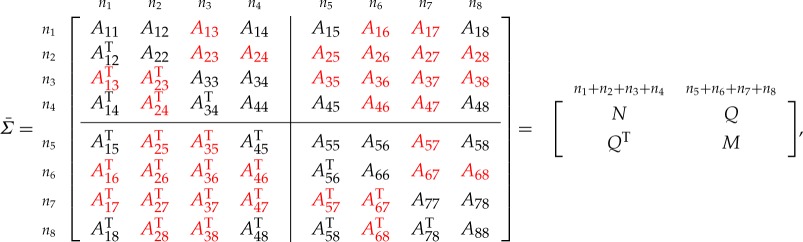

*where the diagonal blocks A*_*ii*_ *are all positive definite, the specified principal minors are all positive and the red blocks*^4^ *are unspecified. The maximal determinant completion of the matrix is*

$$\begin{array}{rl} A_{13} & \;=A_{14}A_{44}^{-1}A_{34}^{T},\;A_{24}=A_{12}^{T}A_{11}^{-1}A_{14},\;A_{23}=A_{24}A_{44}^{-1}A_{34}^{T},\\ A_{57} & \;=A_{58}A_{88}^{-1}A_{78}^{T},\;A_{68}=A_{56}^{T}A_{55}^{-1}A_{58},\;A_{67}=A_{68}A_{88}^{-1}A_{78}^{T},\\ C & \;=DH^{-1}G^{T},\;F=B^{T}A^{-1}D\;\mathrm{and}\;E=FH^{-1}G^{T}, \end{array}$$

*where*

$$\begin{array}{rl} A & \;=\left[\begin{array}{cc} A_{11} & A_{14}\\ A_{14}^{T} & A_{44} \end{array}\right],\;B=\left[\begin{array}{cc} A_{12} & A_{13}\\ A_{42} & A_{43} \end{array}\right],\\ C & \;=\left[\begin{array}{cc} A_{16} & A_{17}\\ A_{46} & A_{47} \end{array}\right],\;D=\left[\begin{array}{cc} A_{15} & A_{18}\\ A_{45} & A_{48} \end{array}\right],\\ E & \;=\left[\begin{array}{cc} A_{26} & A_{27}\\ A_{36} & A_{37} \end{array}\right],\;F=\left[\begin{array}{cc} A_{25} & A_{28}\\ A_{35} & A_{38} \end{array}\right],\\ G & \;=\left[\begin{array}{cc} A_{65} & A_{68}\\ A_{75} & A_{78} \end{array}\right],\;H=\left[\begin{array}{cc} A_{55} & A_{58}\\ A_{58}^{T} & A_{88} \end{array}\right]. \end{array}$$

**Proof.**

**Figure 2.**
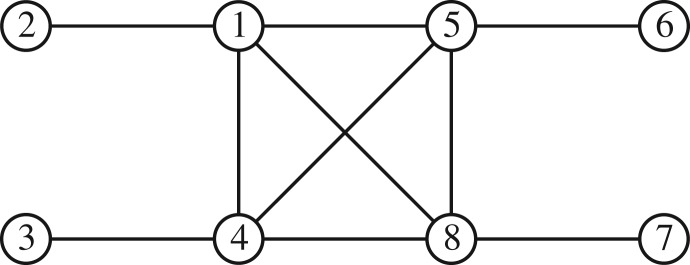
Block chordal graph for matrix $\bar{\Sigma }$ in theorem 6.1, where the numbers refer to the matrix blocks.

First, note that the graph for the matrix $\bar{\Sigma }$ is block chordal, as shown in figure 2, so a positive semi-definite completion exists.

We begin by completing *N* and *M* using theorem 4.1 applied to each block independently, because these do not depend on the corners (the *Q* blocks) as shown by theorem 4.5. Then, having completed the unspecified entries in the diagonal blocks *N* and *M*, we permute $\bar{\Sigma }$ as follows to move the specified blocks within *Q* into the corners, obtaining

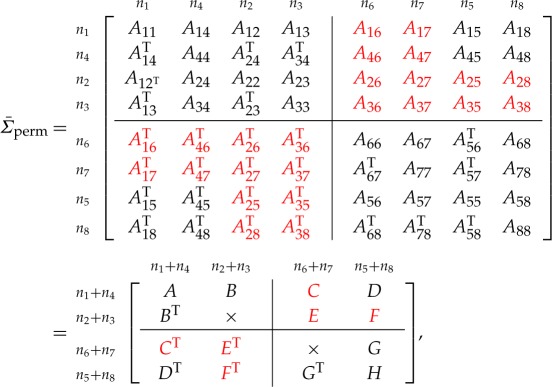

where ‘×’ denotes a block that is not of interest. Finally, we apply theorem 4.1 to solve for the remaining missing entries in the permuted system. ▪

## 7. Concluding remarks

We have derived explicit solutions for completions with maximal determinant of a wide class of partially specified correlation matrices that arise in the context of insurers calculating economic capital requirements. The patterns supported are block diagonal, with either cross-shaped or (inverted) L-shaped gaps on the off-diagonal. The solutions are easy to evaluate, being expressed in terms of products and inverses of known matrices.

Possible directions for future work include developing explicit solutions for more general patterns of unspecified entries and allowing semi-definite diagonal blocks and zero principal minors.

## Footnotes

1 The Matlab code to carry out this experiment can be downloaded from https://gist.github.com/higham/9d9ba7557b1b262c35f6147df8b64eb7.

2 As implemented in the Matlab codes at https://github.com/higham/anderson-accel-ncm.

3 Computed with the Matlab codes at https://github.com/higham/shrinking.

4 The red (unspecified) blocks are *A*_13_,*A*_16_,*A*_17_,*A*_23_,*A*_24_,*A*_25_,*A*_26_,*A*_27_,*A*_28_,*A*_35_,*A*_36_,*A*_37_,*A*_38_,*A*_46_,*A*_47_,*A*_57_,*A*_67_,*A*_68_, and their transposes.

## Data accessibility

This article has no additional data.
